# Traumatic bone cyst of mandible: a case series

**DOI:** 10.1186/s13256-019-2220-7

**Published:** 2019-09-18

**Authors:** Farnoosh Razmara, Zahra Ghoncheh, Ghazal Shabankare

**Affiliations:** 1 Craniomaxillofacial Research Center, Oral and Maxillofacial Surgery Department, School of Dentistry, Tehran University of Medical Sciences, Tehran, Iran; 2 Maxillofacial Radiology Department, School of Dentistry, Tehran University of Medical Sciences, International Campus, Tehran, Iran; 3 School of Dentistry, Tehran University of Medical Sciences, International Campus, Tehran, Iran

**Keywords:** Traumatic bone cyst, Simple bone cyst, Pseuodocyst

## Abstract

**Background:**

A traumatic bone cyst is an uncommon nonneoplastic lesion of the jaws that is considered as a “pseudocyst” because of the lack of an epithelial lining. This lesion is particularly asymptomatic and therefore is diagnosed by routine dental radiographic examination as a unilocular radiolucency with scalloped borders, mainly in the posterior mandibular region. The exact etiopathogenesis of the lesion remains uncertain, though it is often associated with trauma.

**Case presentation:**

We report three Persian cases of traumatic bone cyst with different clinical and radiographic features, and we present a review of the literature to further discuss diagnostic and treatment challenges. Only one of the three patients reported a history of trauma, and despite the usual signs and symptoms of the lesion, extension of the defect to the ramus, swelling of the lingual cortex, and their unusual presence in the anterior mandible were noted in these patients.

**Conclusions:**

Because features of this cyst can be varied, careful history taking and radiographic evaluation alongside the clinical signs and symptoms have a very significant role in definitive diagnosis, appropriate treatment, and accurate assessment of prognosis.

## Introduction

Traumatic bone cyst (TBC) was first described in 1929 as a distinct entity of disease [1]. However, the diagnostic criteria of TBC were not established until 1946. These criteria are still valid and comprise a single lesion without an epithelial lining, surrounded by bony walls and either lacking contents or containing liquid and/or connective tissue [2].

In accordance with the World Health Organization classification, TBCs belong to a bone-related group of lesions in which bony lesions such as aneurysmal bone cyst, fibrous dysplasia, ossifying fibroma, central giant cell granuloma, osseous dysplasia, and cherubism are also included. However, the characteristic that demarcates TBCs from the mentioned true cysts is the absence of epithelial lining, which is why TBCs are regarded as pseudocysts [3].

Aside from being considered as true cysts, TBCs have been referred to in the literature as solitary bone cysts, idiopathic bone cysts, unicameral cysts, simple bone cysts, hemorrhagic bone cysts, primary bone cysts, and extravasation cysts [4]. The TBC is a nonneoplastic intraosseous lesion that mostly affects patients in their second decade of life; the approximate mean age of patients is 20 years [5]. Sex predilection is controversial; although some studies have not found any sex predominance, some have stated a male predilection [6, 7]. The majority of TBCs taking place in the maxillofacial region are preferentially located in the body and symphysis of the mandible; only a few cases in the condylar and anterior regions of the mandible have been reported [8, 9], whereas maxillary lesions are even rarer, but no clear reason explains it [10]. Most patients are asymptomatic, and the lesion is generally discovered incidentally through routine radiographic examination. However, some patients with these lesions may present with pain, swelling, or tooth sensitivity. Fistula, root resorption, pathologic fracture of the mandible, paresthesia, buccal and lingual bony expansion, and delayed eruption of permanent teeth are less common symptoms of this lesion [11].

The etiopathogenesis of TBC remains speculative. On the ground of a widely accepted theory, an unrepaired and disorganized trauma causes a hematoma, which leads to the destruction of the adjacent bone tissue as a result of osteoclastic activity [11–13]. However, this theory does not explain some of the aspects of the lesion, including its enlargement with time, or TBCs of metaphysis and diaphysis of the proximal humerus [14]. The hypothesis of the synovial origin of TBCs was proposed by Mirra *et al.* [15], who explained the development of TBCs as a consequence of a developmental anomaly by which the synovial tissue is subsumed intraosseously. Moreover, Cohen [16] described the lack of a lymphatic drainage of venous sinusoids leading to interstitial fluid entrapment, bony trabecular resorption, and cyst formation as a potential hypothesis. There are some other proposed hypotheses for TBC evolution, including cystic degeneration of primary bone tumors, increased osteolysis, local defects in bone growth, low-grade infection, calcium metabolism disease, ischemic necrosis of bone marrow, intramedullary bleeding, or a combination of these factors [17–19]. However, among the myriad of the mentioned hypotheses, cystic degeneration of tumors, osseous growth abnormality, and a factor triggering hemorrhagic trauma are the three distinguished theories that are proposed [20].

Radiographically, TBC presents as an isolated unilocular radiolucency with a well-defined border that can be either scalloped or irregular [21]. However, multifocal [10] and multilocular [13] cases of TBC have also been reported. When TBC extends to the interdental bone, it illustrates a characteristic radiographic feature called a “scalloping effect” [20]. Confinement of TBCs within the medullary bone rarely exhibits cortical expansion. Comparing the characteristics of TBCs by using computed tomography (CT) and conventional radiography, Suei *et al.* [22] showed that these lesions do not contain air, only liquid. The differential diagnosis must include apical periodontitis, odontogenic keratocyst, central giant cell granuloma, ameloblastoma, odontogenic myxoma, and central and neurogenic neoplasms [23]. Surgery is the management of choice because it ascertains the diagnosis and treatment by generation of a blood clot in the vacant cavity of TBCs and bone regenerates within 6 months [24]. Careful curettage of the lesion usually leads to progressive bone regeneration and a good prognosis, and the recurrence rate is almost negligible [17].

We report three cases of TBC presenting with different clinical aspects, and we further discuss diagnostic challenges of the lesion in this paper. Because these three patients presented with different signs and symptoms, this article can help dentists become familiar with the aspects of this lesion so that they can diagnose and treat it better.

## Case presentations

### Patient 1

**Fig. 1:**
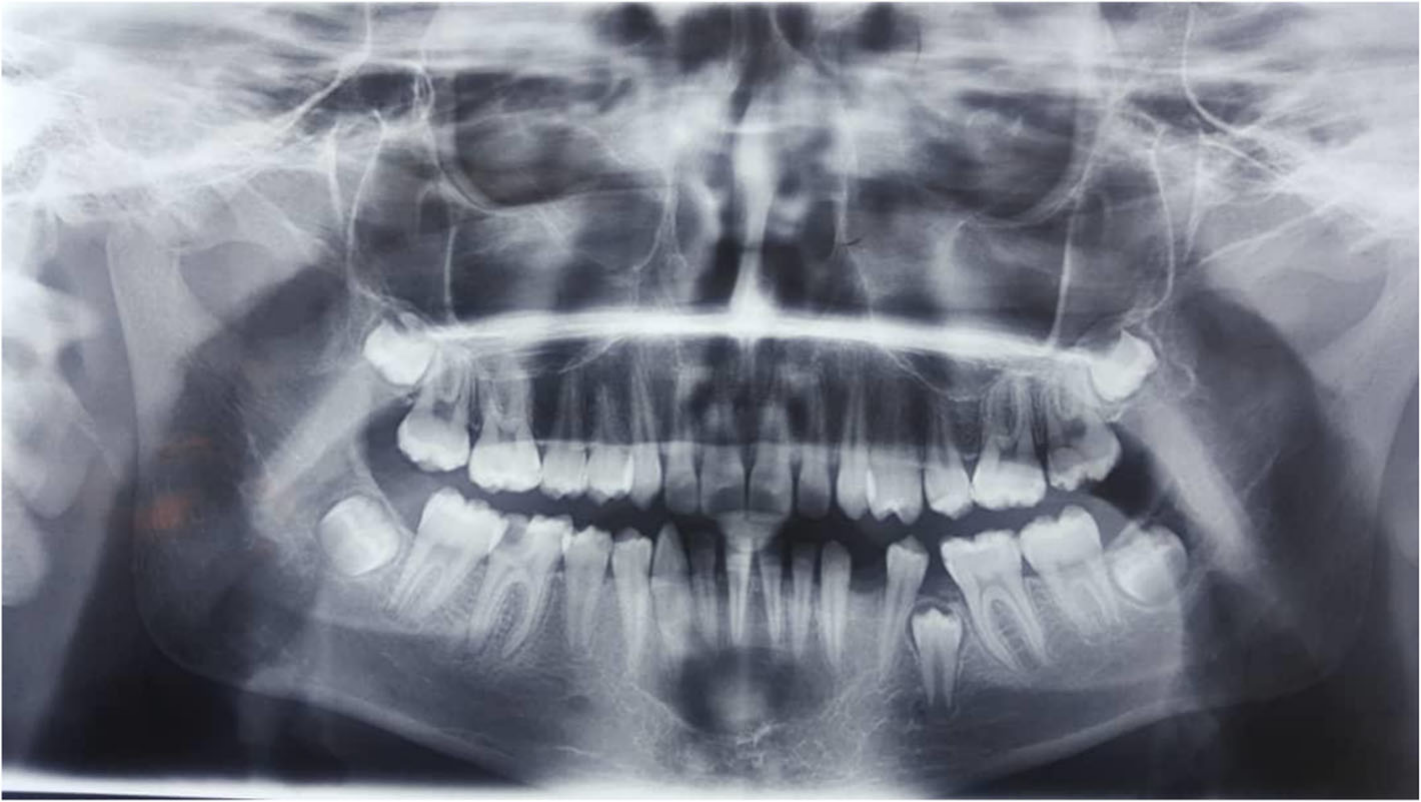
Orthopantomograph revealing extension of the lesion from the distal aspect of the left mandibular canine to the mesial aspect of the right premolar

**Fig. 2:**
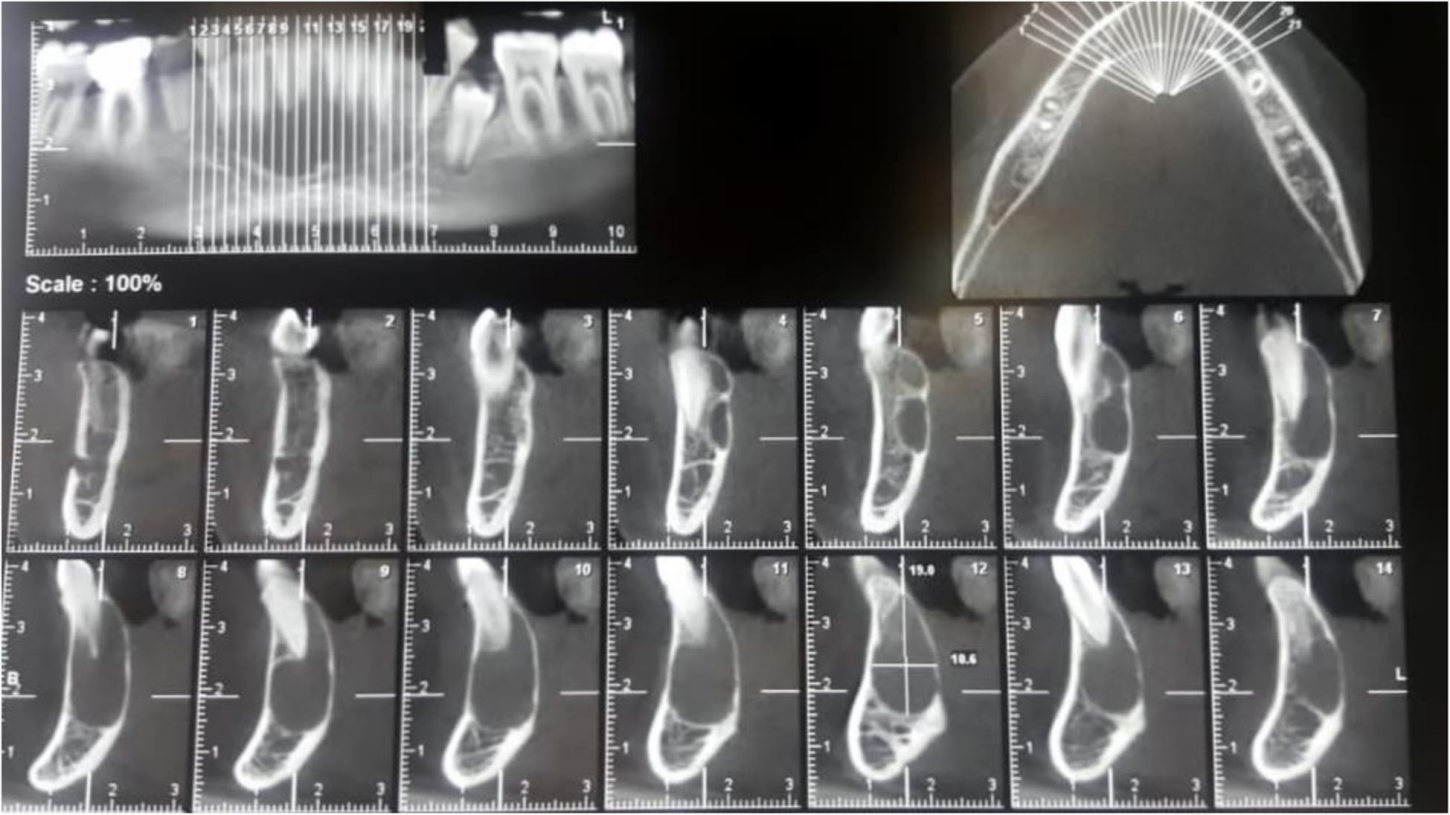
Cone beam computed tomography illustrating a radiolucent area without any buccal or lingual cortical layer perforation or root invasion

**Fig. 3:**
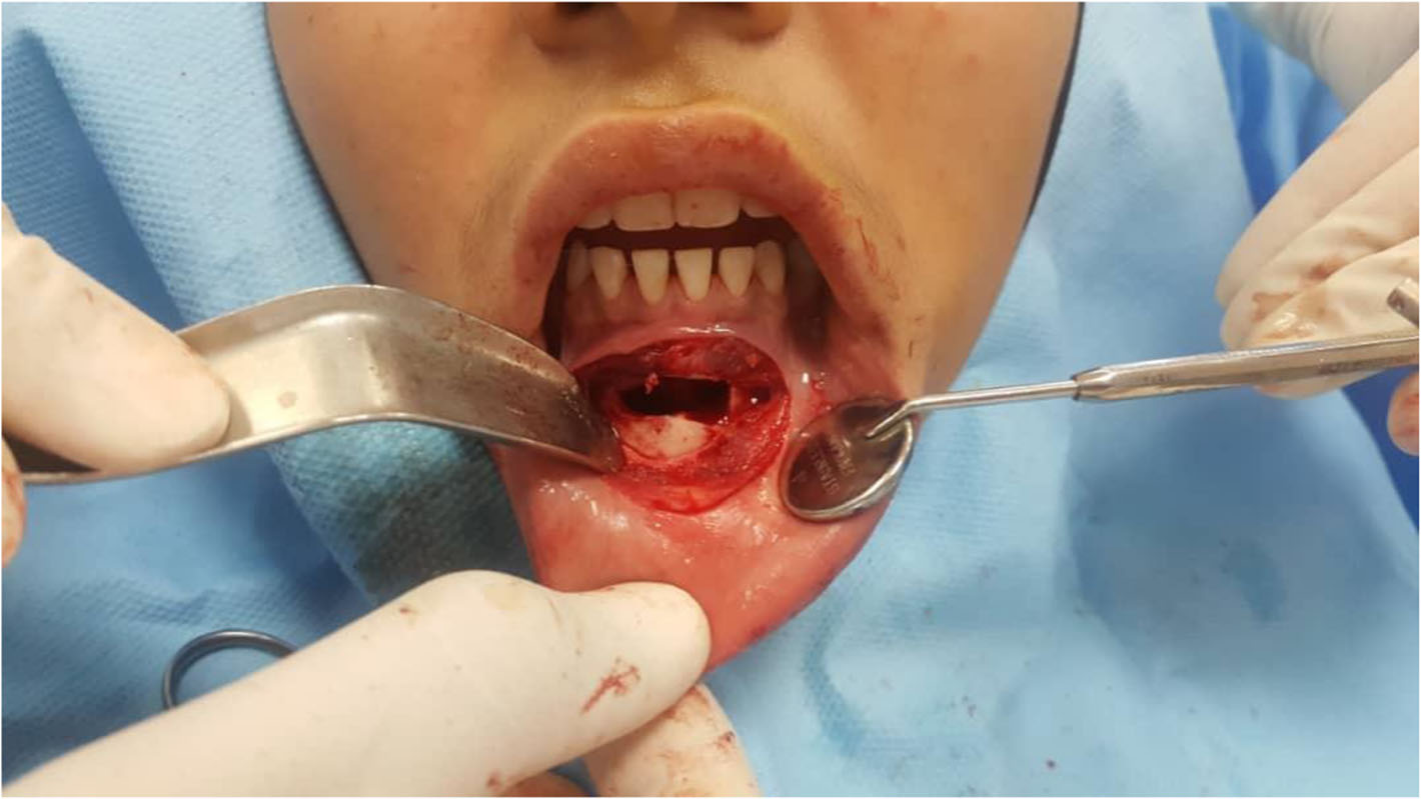
The surgical approach to the lesion. Note the vacant cavity, which is characteristic of traumatic bone cyst

**Fig. 4:**
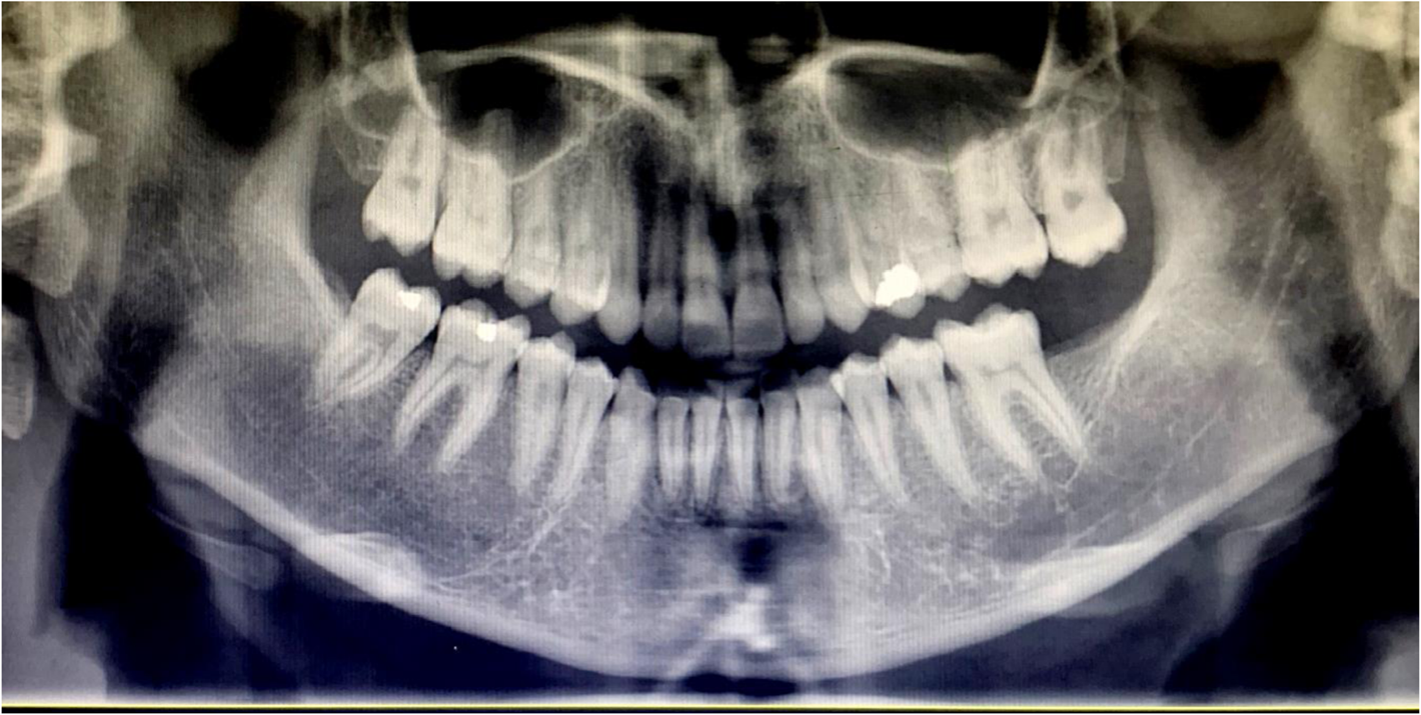
Comparative remission of the cyst 6 months after surgery

A 13-year-old Persian girl with no contributory medical history was referred to a dentist for orthodontic tooth movement. A radiolucent, well-defined lesion was observed by orthopantomography in the anterior mandibular region, which extended to the first premolar area of the right side of the mandible (Fig. 1). The patient did not report a medical condition and did not have smoke or consume alcohol. Moreover, she was receiving no medications before the diagnosis of the lesion. She was then referred to the craniomaxillofacial department of Tehran University of Medical Sciences for further investigation of the lesion. Cone beam computed tomography (CBCT) was ordered, which revealed a well-defined radiolucency with a size of 19 × 10.6 mm in the anterior region of the mandible with no perforation of buccal or lingual cortical layers and no resorption or displacement of the roots. However, slight swelling of the lingual cortex was visible (Fig. 2). A pulp vitality test was performed from the left mandibular canine to the first premolar on the right side, which yielded a positive response. The differential diagnoses were TBC and odontogenic keratocyst. Bilateral mental nerve block was done to anesthetize the surgical site. A sulcal incision was then performed from the left side canine to the first mandibular premolar of the right side, and a full-thickness mucoperiosteal flap was elevated afterward. The surgical approach to the lesion was performed by corticotomy of buccal aspect of the lesion with a round burr, revealing a vacant cavity without an epithelial component, which confirmed the diagnosis of TBC (Fig. 3). The flap was closed with a Vicryl 3-0 suture (Ethicon, Somerville, NJ, USA) after irrigation of the cavity. The patient was then followed in case of progression or relapse of the lesion. The patient reported no complaint during the 6-month follow-up period, and osteogenesis in the defect area was observed (Fig. 4).

### Patient 2

**Fig. 5:**
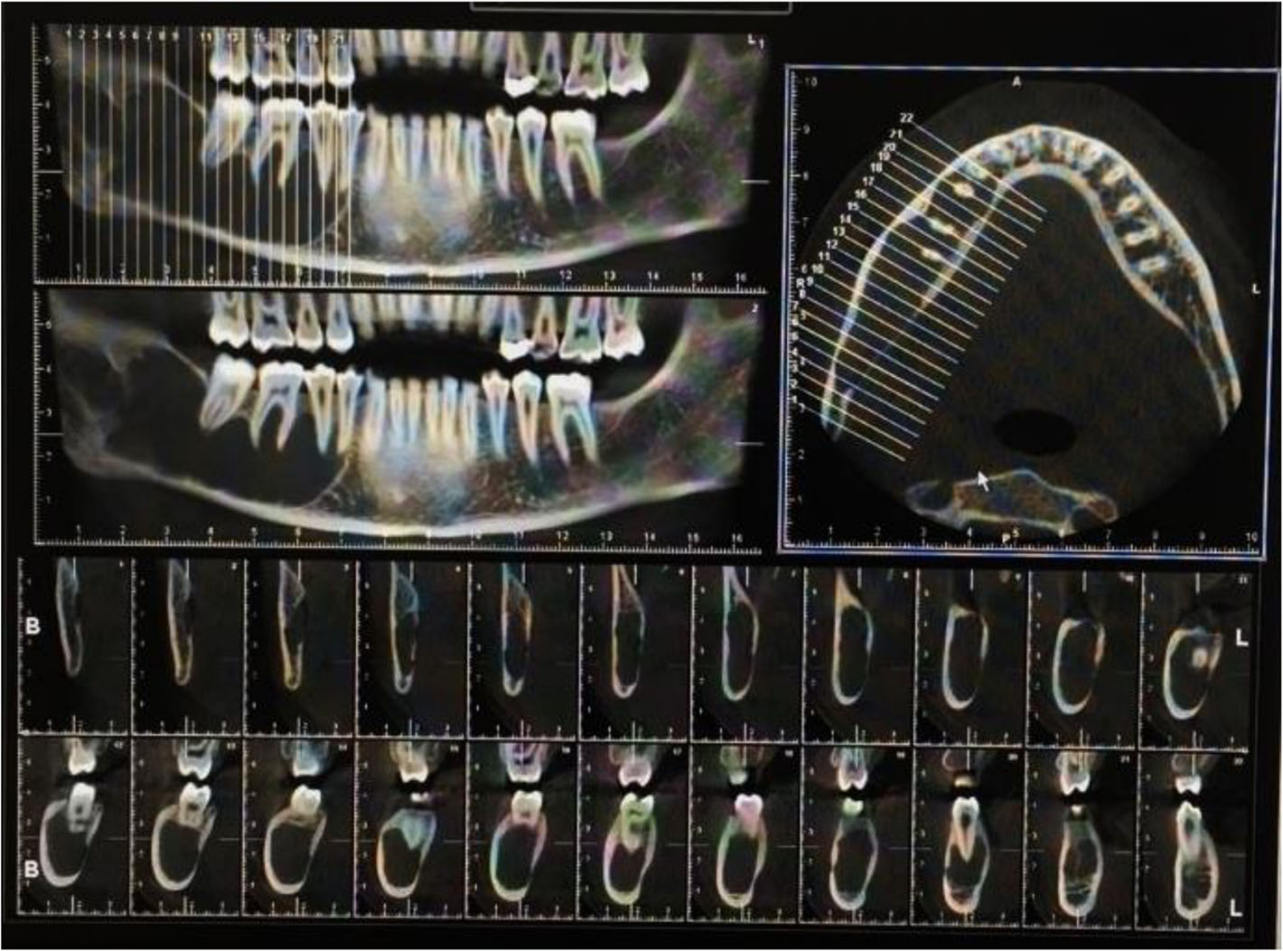
Cone beam computed tomography showing slight expansion of buccal cortex and extension of the lesion beyond the roots of associated teeth

A 14-year-old Persian patient was referred to a dentist with a complaint of mild and numb pain of the right side of her face. The patient reported a history of falling from height in her childhood and no history of a medical condition. The patient did not smoke or consume alcohol and was receiving no medications before the diagnosis of the lesion. Orthopantomography revealed an extensive, unilocular radiolucency that extended from the roots of the first mandibular premolar to the second molar of the right quadrant and the ramus with an overall length of 65 mm. CBCT revealed slight expansion and thinning of the buccal cortex and extension of the lesion beyond the roots. However, no root resorption or displacement was detected (Fig. 5). The teeth were proved to be vital on the basis of pulp vitality test results. The differential diagnoses were TBC and odontogenic keratocyst. A sulcal incision was performed from the right mandibular canine to the posterior region of the mandible. After providing a window approach from the distal aspect of the second molar, aspiration of the lesion was performed, which released bloody fluid. The window approach was then extended, and a blood-containing cavity without an epithelial lining determined the definitive diagnosis of TBC. The patient did not present any complaint during follow-up every 6 months.

### Patient 3

**Fig. 6:**
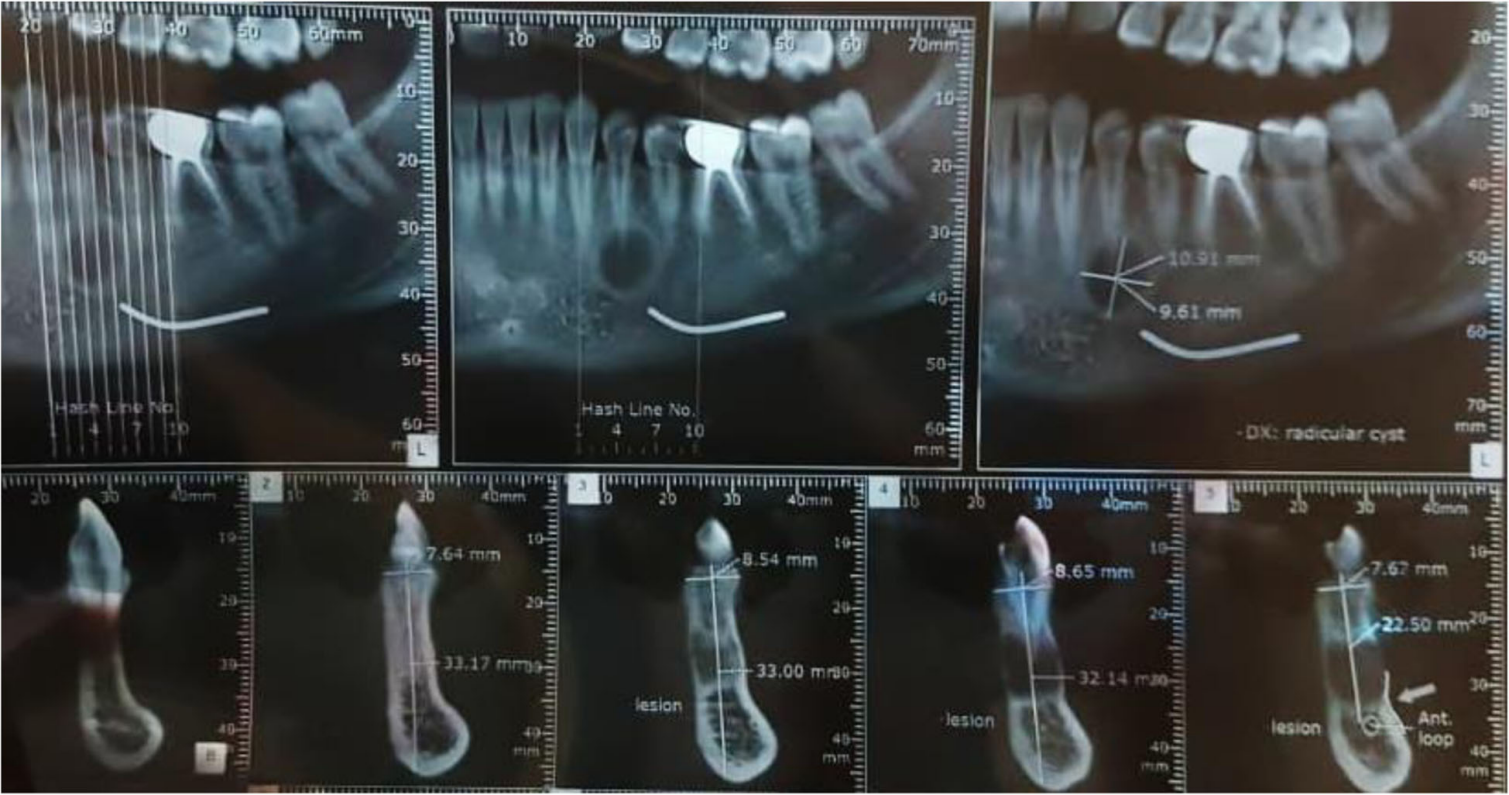
A traumatic bone cyst at the apex of the first mandibular premolar, mimicking a radicular cyst

A 23-year-old Persian woman was referred to a dentist for restorative treatment of the first left mandibular molar. A unilocular lesion mimicking a radicular cyst was accidentally found by orthopantomography at the apex of the first left mandibular premolar. However, because the pulp vitality test did not yield a reliable response, the patient was referred to a craniomaxillofacial surgeon for further examination. CBCT was ordered and revealed a 10 × 9-mm radiolucent lesion at the apex of the first mandibular premolar of the left side with intact buccal and lingual cortical layers (Fig. 6). The patient did not report a medical condition and did not smoke or consume alcohol. Moreover, she was receiving no medications before the diagnosis of the lesion. The differential diagnoses were TBC, cemental dysplasia, and keratocystic odontogenic tumor. A sulcal incision was performed from the left-side mandibular lateral incisor to the second premolar. After full-thickness mucoperiosteal elevation of the flap, corticotomy of the buccal aspect of the lesion, preserving the apex of the root, was implemented. A vacant cavity lacking an epithelial coverage defined the diagnosis of TBC. The patient was followed thereafter and reported no complaints through the 6-month follow-up.

## Discussion

We describe three different cases of TBC, representing varied characteristics of this lesion. TBC has been referred to by different names in the literature. When occurring in the jaw, traumatic, hemorrhagic, or extravasation bone cysts are the preferred diagnostic terms. However, extragnathic lesions are usually termed simple, solitary, or unicameral cysts [25, 26]. The wide variety of names is indicative of the etiology of the lesion as a matter of conjecture. Although the traumatic-hemorrhagic hypothesis is widely accepted beyond the divergent views regarding the etiology of TBC, it does not agree with development of the lesion without a clear history of trauma to the orofacial region in many cases [27]. Moreover, the incidence of history of trauma in patients with TBC is not greater than that in the general population and is wide-ranging, from 17% to 70% on the basis of reported case series [11, 28]. Additionally, men display a higher incidence of trauma, and the anterior mandibular region is predominantly traumatized, whereas TBC is equally dispersed between sexes and occurs in posterior regions of the mandible [29]. Hence, the relevance of trauma to development of TBC is open to question. Among the three cases discussed in the present article, only one had a clear antecedent of trauma.

TBC is related to some medical conditions in some articles. Pogrel reported a case of solitary bone cyst, possibly related to impacted third molar extraction [30]. In another report of a rare case, Nagori *et al.* [31] suggested a possible correlation between Langer-Giedion syndrome and multifocal TBC.

TBCs are commonly located in the mandibular body, above the inferior alveolar canal [21]. Predominantly, they appear in the posterior region and may extend from the canine to third molar area [32]. Ascending mandibular ramus and chin symphysis are other possible but less common sites of TBC development [17]. TBCs are barely found in the maxilla, and if they occur, unlike their mandibular counterparts, they appear in the anterior region [33]. However, it is conceivable that the radiographic visualization of maxillary lesions is more challenging because of the presence of the maxillary sinus [34].

TBCs mostly occur in the second and third decades of life and have a slight male predominance or no gender predilection [35]. Olech *et al.* [13] hypothesized that TBC is the result of a failure in early organization of a hematoma in marrow spaces. This theory is in harmony with the fact that TBCs occur in young individuals more commonly. The trauma-related and self-healing nature of TBCs can provide another explanation for their increased incidence in the young population [36, 37]. However, reports of younger and older patients have been registered [32, 38].

TBC is predominantly an asymptomatic lesion. In morbidity cases, however, pain can be the most significant symptom in 10% to 30% of the patients [39]. On the basis of Howe’s study, swelling might be a presenting symptom in 27% of the cases [18]. Although rare, expansion of the buccal cortical layer may occur, resulting in intraoral or extraoral swelling, but it seldom leads to facial deformity [40]. However, swelling can be the chief complaint of patients with TBC [41]. Paresthesia of the lower lip or chin is also a rare but possible chief complaint of patients with TBC that is representative of mandibular nerve neuropathy [42]. The adjacent teeth tend to remain vital in 85% of cases, and nonvitality of the teeth neither results in development of the lesion nor is a result of the lesion [43]. There is not any increased tooth mobility or color change of abutting teeth. The teeth are barely sensitive to percussion [44]. Reports have been published of inferior alveolar canal displacement to the inferior border of the mandible or to the lingual cortex [40, 41].

On imaging, TBC usually presents as a radiolucent, unilocular, well-defined, isolated lesion with irregular or scalloped borders that often suggest the diagnosis. Extended to the interdental bone spaces, TBCs present a festooned, scalloped, or lobular pattern [26]. The radiographic appearance of our cases is in good harmony with the mentioned features.

However, the radiographic appearance of TBCs may not match the usual characteristics discussed above in this article. Kuhmichel and Bouloux reported an unusual presentation of multiple unicystic TBCs in the symphyseal region of a female patient [10]. According to a review of 161 cases, multiple synchronous lesions may occur in 11% of the cases [28].

Radiographic presentation of TBC might have some features in common with some other lesions, alongside confusing signs and symptoms, so that it can lead to an inappropriate diagnosis and treatment plan in some cases. TBCs can mimic a radicular cyst if placed at the apexes of teeth or a keratocyst odontogenic tumor because of the small expansion of cortical layers and scalloped borders. A history of trauma and vital associated teeth are features that elevate the possibility of TBC diagnosis in young patients with radiolucent lesions. CT is considered to be an applicable diagnostic tool for initial diagnosis [45].

Interestingly, Suei *et al.* [46] asserted that radiographic features of TBCs can be beneficial for forecasting the possible prognosis as well as for diagnosis and discovery of the lesion. According to the results of their study, lesions with intact lamina dura heal either after treatment or spontaneously because an unbroken lamina dura is a sign of probable healing after treatment. Moreover, the presence and nature of an expansion is another contributing factor in the prognosis of the lesion. Lesions without an expansion or with a smooth nature of expansion tend to heal, whereas lesions with nodular expansion have a tendency to recur. Additionally, multiple lesions or those with osseous dysplasia are remarkably associated with a high rate of reoccurrence.

Treatment methods of TBC are varied, and each technique has a different rate of recurrence, except for the complete resection of the cyst [47]. Complete curettage and bone grafting are the most common and useful methods of treating these lesions [48]. Çelik *et al.* [49] suggested that inadequate curettage of the cyst can lead to recurrence as a result of residual tumor tissues. They also emphasized the importance of sufficient curettage for pediatric patients because the residual tumor tissue’s proximity to the physeal line is associated with the aggressive development of the cyst in children.

However, spontaneous resolution of TBCs is possible in some untreated cases. Although the surgical approach is considered a secure way of diagnosis and treatment of these lesions, follow-up without surgical intervention can be a choice for selected cases based on their epidemiological, clinical, and radiographic features. Availability of the patient for long periods of follow-up is of great importance when a nonsurgical protocol is chosen [50].

Newton and Zunt [51] suggested that endodontic treatment of involved teeth in TBC cases should be considered before, during, or after surgical treatment of the lesion. Endodontic treatment of adjacent teeth with compromised pulp vitality can be implemented prior to the surgical procedure because it eliminates the potential focus of inflammation, which leads to necrosis of the pulp. When an accurate vitality test result cannot be achieved, a test cavity and pulp exposure may be indicated [51].

Other treatment modalities have been carried out as well. Subramanian *et al.* [52] claimed that careful curettage and the use of plasma-rich protein as a means of bone regeneration lead to faster favorable healing and are safe for use in children. In another study, Aiba *et al*. [53] suggested endoscopic curettage as a minimally invasive method for treating aneurysmal bone cysts with a recurrence rate of 10%, which is comparable to that of other procedures. Other treatment approaches, such as application of methylprednisolone acetate, bone allografts, and insertion of gelfoam saturated with penicillin and thrombin are also tried [54]. Obliteration of the cavity by bone formation after surgical exploration is commonly rapid and may take 3 to 12 months [11].

## Conclusion

We describe clinical and radiographic features of three cases of TBC. Because features of this cyst can be varied, careful history taking and radiographic evaluation alongside the clinical signs and symptoms have a very significant role in definitive diagnosis, appropriate treatment, and accurate assessment of prognosis.

## Data Availability

The datasets used and/or analyzed during the current study are available from the corresponding author on reasonable request.
